# Uniform electric field generation in circular multi-well culture plates using polymeric inserts

**DOI:** 10.1038/srep26222

**Published:** 2016-05-19

**Authors:** Hsieh-Fu Tsai, Ji-Yen Cheng, Hui-Fang Chang, Tadashi Yamamoto, Amy Q. Shen

**Affiliations:** 1 Micro/Bio/Nanofluidics Unit, Okinawa Institute of Science and Technology Graduate University, Okinawa, 904-0495, Japan; 2 Research Center for Applied Sciences, Academia Sinica, Taipei, 11529, Taiwan; 3 Cell Signal Unit, Okinawa Institute of Science and Technology Graduate University, Okinawa, 904-0495, Japan

## Abstract

Applying uniform electric field (EF) *in vitro* in the physiological range has been achieved in rectangular shaped microchannels. However, in a circular-shaped device, it is difficult to create uniform EF from two electric potentials due to different electrical resistances originated from the length difference between the diameter of the circle and the length of any parallel chord of the bottom circular chamber where cells are cultured. To address this challenge, we develop a three-dimensional (3D) computer-aided designed (CAD) polymeric insert to create uniform EF in circular shaped multi-well culture plates. A uniform EF with a coefficient of variation (CV) of 1.2% in the 6-well plate can be generated with an effective stimulation area percentage of 69.5%. In particular, NIH/3T3 mouse embryonic fibroblast cells are used to validate the performance of the 3D designed Poly(methyl methacrylate) (PMMA) inserts in a circular-shaped 6-well plate. The CAD based inserts can be easily scaled up (i.e., 100 mm dishes) to further increase effective stimulation area percentages, and also be implemented in commercially available cultureware for a wide variety of EF-related research such as EF-cell interaction and tissue regeneration studies.

A weak direct-current electric field (dcEF) exists at the tissue level due to the transepithelial potential difference established by the tissue polarity^1^. Cells demonstrate directional migration (electrotaxis) or orientation-change (electro-alignment) in response to a physiological dcEF in both *in vitro* and *in vivo* settings. The electrotaxis and dcEF stimulation have played pivotal roles in physiological processes such as embryonic development, neurogenesis, morphogenesis, and wound healing^1^,^2^,^3^,^4^,^5^.

Numerous cellular signalling pathways have been regulated under electric field (EF) stimulation. Various membrane receptors^6^,^7^,^8^,^9^,^10^ or ion channels^11^,^12^,^13^,^14^,^15^ have been suggested to act as EF sensors and initiate many intracellular signalling cascades in different cell types^8^,^13^,^16^,^17^,^18^,^19^,^20^,^21^. Further investigations are required to clarify the functional roles of EF sensory proteins and signalling networks in regulating the electrotaxis phenomena.

Gaining a better understanding of signalling pathways demands a reliable and convenient electrical stimulation platform for microscopy imaging and cell product recovery with subsequent biochemical analysis. Even though an electrical cue can direct cell migration comparable to that of chemical cues^22^ and synergistically promote directional migration with other physical factors such as shear stresses^23^, electrotaxis is less well studied than chemotaxis, possibly due to the lack of experimental tools for convenient EF stimulation comparable to a boyden chamber (transwell chamber) that is routinely used for chemotaxis^24^.

Conventional *in vitro* electrical stimulations were commonly performed either by direct stimulation using electrodes, or stimulation in a microfluidic chamber with salt bridges. The EF created through direct electrode stimulation is not uniform and cells are often exposed to toxic electrolysis products. Thus conventional electrotaxis studies usually employ a confined microfluidic chip in which cells are cultured in the bottom of the culture chamber^25^,^26^,^27^,^28^,^29^,^30^. The small cross-section of the chamber limits the applicable electrical current and reduces the Joule heating that could be harmful to the cells.

Despite the success of using microfluidic chips for electrical stimulation in recent studies, these microfluidic chips often require special fabrication procedures on cell culture dishes days prior to the actual experiment, limiting the adaptivity with common laboratory settings. Further, a simple rectangular shaped cell culture microchamber is usually placed on a circular shaped tissue-culture polystyrene (TCPS) petri dish to generate the uniform EF. As a result, a large portion of the cell culture area on the dish is unused, leading to low cell yield and poor cell product recovery. Even though larger cell yields have been recently achieved by scaling up the rectangular shaped microchamber with increased cell culture area^29^,^30^, a large fraction of the circular shaped TCPS dish is still unutilised. In a circular-shaped area, a uniform EF cannot be intuitively created by two electric potentials due to different electrical resistances originated from the length difference between the diameter of the circle and the length of any parallel chord of the bottom circular chamber where cells are cultured. For example, Marotta *et al*. electrically stimulated muscle cells to pace contraction by using a 6-well plate^31^. The cells were subjected to non-uniform EF as well as electrolysis products (see Supplementary Fig. S1). Tissue pacing studies with a commercial electrical stimulation system suffered similar drawbacks (C-dishes, IonOptix, MA, USA)^32^,^33^,^34^,^35^ (see Supplementary Fig. S2). Lin *et al*. used a modified transwell assay to study cell electrotaxis by applying EF through the transwell insert coupled with platinum electrodes^36^. Alternatively, Garciá-Sánchez *et al*. used patterned electrodes to stimulate cells in multi-well plates^37^. Their systems not only require sophisticated microfabrication procedures but small EF-null gaps between electrodes also decrease the EF homogeneity. Recently, Ahirwar *et al*. used eletromagnetic induction method with a boyden chamber to demonstrate non-contact directing electrotaxis, but non-uniform EF persisted^38^.

Computer aided design and computer aided manufacturing (CAD/CAM) use computer software to precisely design model structure and program manufacturing processes. Mathematically depicted 3D structures for workpieces can be easily created by CAD/CAM software and conveniently adopted for numerical simulations. Thus time, material, and manpower are greatly reduced for prototyping effort. In recent years, additive manufacturing (3D printing)^39^ takes the advantage of CAD/CAM to rapidly prototype workpieces through layer-by-layer stacking of raw materials and this technology has been used to fabricate microfluidic chips^40^,^41^,^42^,^43^.

In this study, we solve the non-uniform EF challenge by applying the CAD principle to modulate the electrical resistance in a polymeric insert, which is then retrofitted to a common multi-well plate. Using Ohm’s law, an optimised CAD structure is created to equalize the electrical resistance in the circular shaped bottom chamber to generate a uniform EF. As a result, a large area of contemporary cell culture dish can be stimulated with the uniform EF and higher cell yield is obtained.

## Materials and Methods

### Microfluidic chip composition and assembly

**Figure 1:**
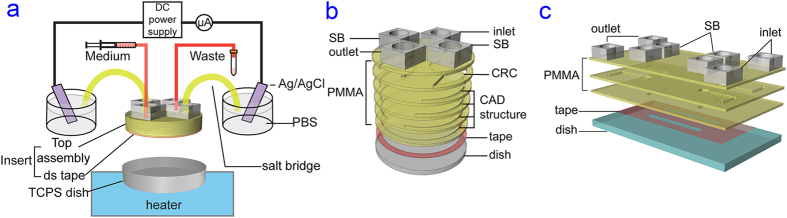
(**a**) The schematic diagram of electrical stimulation setup by using the 6-layered PMMA insert for uniform EF stimulation on cells. (**b**) Schematics of the layered insert (not to scale). PMMA top assembly containing current rectifying chambers (CRC) and 3D CAD structures are affixed to the cell culture dish through a piece of double sided tape to form the assembled microfluidic chip. Salt bridge is abbreviated as SB. (**c**) Schematics of the simple rectangular channel chip (not to scale). The PMMA containing a world-to-chip interface was affixed to the cell culture dish through a piece of double sided tape containing two rectangular channels to form the assembled microfluidic chip.

The schematic diagram of the electrical stimulation setup by using an assembled microfluidic chip is shown in Fig. 1a. The entire microfluidic assembly consists of two main compartments. The bottom structure is a standard tissue culture polystyrene (TCPS) dish (shown in grey) that was mounted on an indium tin oxide (ITO) transparent heater on the microscope stage for temperature control required for cell culture operation^44^. The insert part (green region with grey top) is affixed to the TCPS dish by a double sided tape (red) adhered to the inner side of the dish (see Fig. 1b^30^). The polymeric insert is fabricated in layered Poly(methyl methacrylate) (PMMA) based on 3D computer-aided design to provide current rectifying chambers (CRC) and accommodate inlet, outlet, and salt bridges (SB) tubing interfaces. To validate the performance of the insert for electrotaxis, a simple microdevice with rectangular channels was fabricated and used as a control platform (Fig. 1c). Two identical rectangular channels (30 mm × 3 mm × 0.07 mm, L × W × H) were designed in a single chip. Similar channel dimensions have been used to study the electrotaxis of many cell lines^28^.

### Design principle

**Figure 2:**
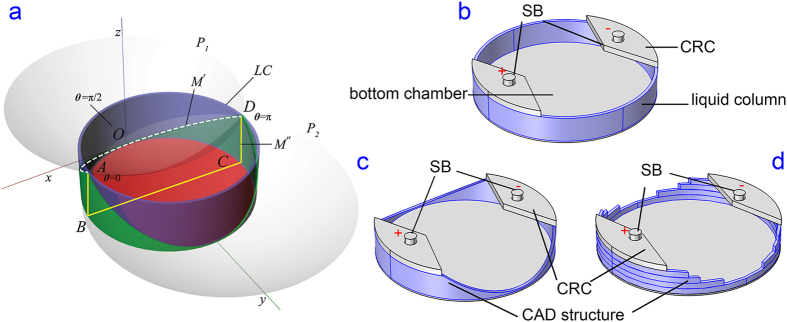
Computer aided design principle. (**a**) The cell culture area is shown in red in the bottom chamber; the CAD structure of the top assembly is shown in green, which is formed by intersecting the liquid column (*LC*, purple) with two paraboloids (*P*_1_ and *P*_2_, grey). (**b**) A 3D model of a plain polymeric insert without the 3D CAD structure. The liquid column is shown in blue. Salt bridges are abbreviated as SB. Current rectifying chambers are abbreviated as CRC. (**c**) A polymer insert model with a smooth 3D CAD structure (blue) to be inserted in a 6-well plate. (**d**) A 3D model of layered insert (in blue) design to approximate (**c**).

A uniform EF cannot be simply created in a circular area due to different electrical resistances originated from the length difference between the diameter of the circle and the length of any parallel chord. Our goal is to create uniform EF in a circular shaped chamber with the largest possible surface area for cells. As illustrated in Fig. 2a, cells occupy the red region of the bottom chamber (*xy* plane with diameter 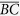, with its centroid on the *y*-axis). The insert on top of the red region contains a thin liquid column (*LC*, purple, Fig. 2a), residing on top of the bottom chamber. The assembled microdevice contains the bottom chamber, liquid column, and the current rectifying chambers (CRC, also termed as world-to-chip interface), see Fig. 2b. An electrical current flowing from one salt bridge (i.e., *A*) to the other (i.e., *D*) cannot create a uniform EF in the bottom chamber (see more simulation details in the results section). To create a uniform EF, the electrical resistance from one salt bridge to the other through any cross-section in the system must be the same and all the electrical currents must pass through the bottom chamber uniformly.

To address this challenge, we designed a three dimensional CAD structure to equalize the electrical resistance through any arbitrary current path that passes through the bottom circular chamber. The structure is shown as green in Fig. 2a and obtained from intersecting the liquid column (*LC*) by two identical circular paraboloids (*P*_1_ and *P*_2_) whose apexes are located at the intersects of the midline of the 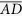 and the liquid column *LC* (Fig. 2a). The apex *O* of the paraboloid *P*_1_ coincides with the origin of the coordinate system. The paraboloid intersects with the liquid column at the curve 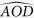 (white dashed curve, Fig. 2a) where the projections of points *A* and *D* on *xy* plane are *B* and *C*. The electrical resistance can be calculated by considering both the length and the cross-sectional area according to Ohm’s law^45^. To facilitate a uniform EF in the bottom chamber, the electrical resistance passing through curve 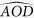 (see Fig. 2a) must be equal to that through 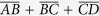 (yellow lines in Fig. 2a). In other words, the electrical current passing along the parabolic curve 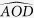 should hold the same strength to the electrical current passing through 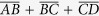.

The arc length of curve 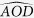 can be determined by using multivariate calculus^46^, see detailed derivation and the design principle in Section 2 of the SI document. In short, the constant of the paraboloids describing the level of curvature in *xz* and *yz* planes and the height of the liquid column can be calculated and used for model design in a commercial CAD software package (Rhinoceros, USA). The designed CAD structure is illustrated in Fig. 2c.

### Numerical EF simulation

The CAD model for a plain polymeric insert and the CAD model created by the aforementioned principle was imported into COMSOL Multiphysics software (COMSOL Inc., USA). The model used the culture medium (Dulbecco’s minimum essential medium, DMEM) as the ionic fluid and the electric potential between the salt bridges was numerically simulated by solving steady-state Maxwell’s equations using the alternating current/direct current (AC/DC) module in COMSOL (Fig. 2b,c). The conductivity of DMEM was measured to be 1.515 S/m (F74 with 3553-10D conductivity probe, Horiba, Japan, see Supplementary Fig. S3) and this value was input in the COMSOL. A current density of 376.1 A/m^2^ aimed to create 100 mV/mm of EF was set as the boundary condition at one salt bridge connection, and a ground potential was set for the other salt bridge (Fig. 2c). The electric field strengths (EFSs) at the bottom of the cell culture chamber were analyzed to assess the EF uniformity at a height of 10 μm. The EFS data points at positions where the liquid column resides were excluded. The numerical simulation results were exported and analyzed in Prism 6 software (Graphpad, USA).

Due to the limitation of in-house fabrication tools, the assembled microfluidic chip based on the 3D CAD model with smooth paraboloid surface was not fabricated in this work. Instead, an approximated 6-layered model (Fig. 2d) compatible with PMMA thermoplastic manufacturing procedure was applied. This 6-layered PMMA insert possessed the same thickness (6 mm) as the original plain 3D CAD model and the same paraboloid parameters were employed in the fabrication. Based on this proof of concept study, the 3D CAD insert can be easily manufactured by computer numerical control (CNC) manufacturing technologies in the future. To examine the robustness of creating the uniform EF by using layered inserts, the tolerance of the EFS and uniformity to different cell chamber thickness ranging 0.2–0.3 mm was also simulated.

### Device fabrication and EF measurements

The 6-layered approximation model for both 6-well plates and 35 mm dishes was used for the fabrication of inserts. Patterns were designed in AutoCAD software (Autodesk, USA) and a 1 mm thick PMMA substrate (Comoglas, Kuraray, Japan) was cut based on the 3D design by using a CO_2_ laser cutter (VLS2.30, Universal Laser Systems, USA). The layers were aligned and joined by thermal bonding and polymeric tapes (Fig. 1b). The detailed fabrication procedure was previously reported^47^,^48^. Adapters for fitting connection were super-glued onto the inserts (406 Prism Instant Adhesive, Loctite, USA). The double sided tape for the insert was then affixed to the insert bottom (0.26 mm-thick, F9473PC, 3 M, USA). The fabrication process of the simple rectangular channel chip followed the same procedure as those for the circular insert.

To measure the EF in the bottom chamber in the insert, an array of holes in 0.3 mm diameter were drilled on the 1 mm-thick PMMA substrate^25^,^28^. The spacing between each hole was 3 mm. The holes were temporarily sealed with a Kapton tape. The insert was filled with Dulbecco’s minimum essential medium (DMEM, 12800017, Gibco, USA). An 46 V electric potential was applied through Ag/AgCl electrodes (25 mm × 100 mm) by a DC power supply (E3641A, Keysight technologies, USA). Preparation of Ag/AgCl electrodes was described elsewhere^30^. To measure the voltage differences, two Ag/AgCl wire based electrodes (0.3 mm in diameter) were inserted into two adjacent holes after piercing the tape cover (see Supplementary Fig. S4)^30^,^44^. The voltage differences between any two electrodes in the chamber were measured by a digital multimeter (2100, Keithley Instruments, USA) for 20 samples at every position by using the Excel add-in function provided by the manufacturer (KI-LINK, Keithley Instruments, USA). The EFSs can then be calculated by dividing the voltage differences by the distance between respective electrodes. The results of mean EFSs and standard deviations are calculated and exported using a custom MATLAB script (Mathworks, USA), see Table 1.

### Cell culture and maintenance

A Swiss murine embryonic fibroblast cell line with 3-day transfer protocol, NIH/3T3 (American Type Culture Collection, ATCC, USA), was used in this study to demonstrate the electrical stimulation functionality with the polymeric circular insert and the rectangular microchannel. The cells were cultured on TCPS dishes in DMEM supplemented with 10% fetal bovine serum (FBS, Sigma-Aldrich, USA) at 37 °C in a 95% relative humidity atmosphere supplemented with 5% CO_2_. The cells were sub-cultured twice a week by the recommended split ratio with trypsin-EDTA (Life Technologies, USA). For long term storage, the cells supplemented with 10% dimethylsulfoxide were cryopreserved in liquid nitrogen.

### EF stimulation and microscopy analysis

**Figure 3:**
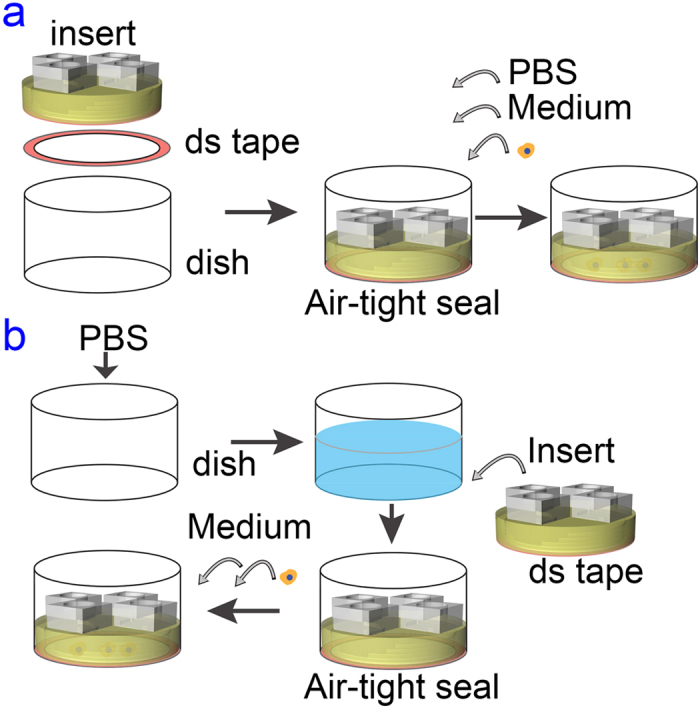
The workflow of the cell experiment by using the polymeric insert. (**a**) The insert was affixed to the dish first. PBS, cell culture medium, and cell suspension were infused into the microfluidic chip sequentially. (**b**) To further avoid bubble entrapment, the insert can be affixed to the dish in PBS. Thereafter the buffer is replaced by cell culture medium and cell suspension is then inoculated.

Each six-layered PMMA insert was disinfected and then affixed to individual wells in a 6-well TCPS plate or to a 100 mm TCPS dish (see Fig. 1b). Similar procedure was applied to the simple rectangular channel chip. To avoid entrapment of bubbles, which could disrupt EF uniformity and cause cell death, assembled microfluidic chips were primed by CO_2_ gas, and filled with phosphate buffered saline (PBS, Life Technologies, USA) as shown in Fig. 3a. Alternatively, the inserts can be affixed to the well bottom with the presence of PBS as shown in Fig. 3b to further reduce bubble entrapment because the double sided tape has a limited adhesiveness in protein-free buffer solution.

To start the cell experiment, PBS pre-filled chamber was first replaced by serum-containing cell culture medium, and a suspension of 5 × 10^5^ cells was subsequently loaded into the chamber through the salt bridge ports by gravity feeding. After overnight culture for cell adhesion and growth, fittings to supply culture medium and for salt bridges (containing 1.2% agarose (LE agarose, Lonza, USA) in PBS) were connected to the top of the inserts. A syringe pump (YSP-202, YMC, Japan) was used to exchange cell culture medium during the time lapse experiment at a flow rate of 100 μL/h for the circular insert and 20 μL/h for the rectangular channels to obtain similar shear stress acting on the cells. A DC voltage and the current was applied and measured by a high voltage source meter unit (2410, Keithley Instruments, USA) through Ag/AgCl electrodes in PBS. The required current for a 300 mV/mm EF in a chamber of 30 mm in diameter and 0.26 mm in thickness was 3.545 mA. The EF stimulation setup diagram and a snapshot of the 6-well plate is shown in Fig. 1a and Supplementary Fig. S5.

The time lapse electrotaxis experiments were carried out on an automated microscope (Ti-E, Nikon, Japan) (Supplementary Fig. S6). The phase contrast cell images were taken at different positions across the devices at an interval of 5 minutes. The morphology and centroid of cells were tracked manually for the duration of 5 hour time lapse using ImageJ analysis software package. All data are represented as the mean ±95% confidence interval, which is 1.96 of standard error of mean, from triplicate experiments. Kruskal-Wallis one-way analysis of variance on ranks test with Dunn’s multiple comparison post-hoc test were performed when non-Gaussian distribution of sample data was obtained from Bartlett’s test. The confidence level to reject a null hypothesis between two data sets was set at 95%. A p-value (the probability for a true null hypothesis) less than 0.05 represents a statistical significance at 95% confidence.

## Results and Discussion

### 3D CAD optimisation for uniform EF creation

The current density at the bottom of the chamber was simulated for the plain polymeric insert, the smooth polymeric insert with the 3D structure designed to intersect a liquid column by paraboloids, and the layered approximation for the PMMA insert (see schematics in Fig. 2(b–d)). With the liquid column thickness of 0.5 mm, uniform EF can be obtained for a 0.26 mm thick chamber using a 6 mm thick insert. A 6 mm thick insert can also be designed for a 0.13 mm thick chamber by decreasing the liquid column thickness to 0.25 mm.

**Figure 4:**
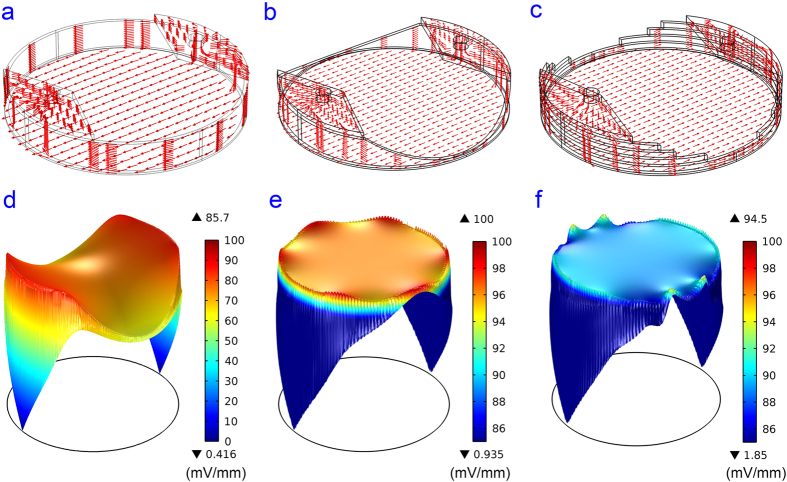
COMSOL simulation results. (**a**) The current density in a plain polymeric insert; (**b**) The current density in a smooth 3D CAD insert; (**c**) The current density in a layered PMMA insert; (**d**) The EFS at the bottom of the chamber with a plain polymeric insert; (**e**) The EFS at the bottom of the chamber with a smooth 3D CAD insert; (**f**) The EFS at the bottom chamber with the layered PMMA insert.

Next we show side-by-side comparisons of simulated EF results for plain polymeric inserts, smooth 3D CAD inserts, and layered 3D CAD inserts (see Fig. 4). Without the 3D designed structure, a large portion of the electrical current (in red) passes through the liquid column instead of passing through the bottom chamber where the cells are located (Fig. 4a) and creates a non-uniform EF (Fig. 4d). With the 3D designed structure, the current lines (in red) are uniformly distributed in the bottom chamber, indicating that a uniform and directional EF was created (Fig. 4b). The highly directional EF created by using the insert also suggests that the inserts are suitable not only for cell stimulation but also for electrotaxis studies. Figure 4e shows the uniformity of the EFS in the smooth 3D CAD insert. A uniform decrease in the electric potential in the bottom chamber can be found in Supplementary Fig. S7. Figure 4c,f exhibit a uniform and directional EF being created by the approximated layered PMMA insert.

The simulated results are summarised in Table 1. In a plain polymeric insert without the 3D designed structure, the EF is non-uniform and the mean EFS and coefficient of variation (CV, defined as the ratio of the standard deviation to the mean), are 75.03 mV/mm and 7.91%. In the 6-well plate (or a 35 mm dish) with our designed insert, an EF with mean EFS of 96.1 mV/mm is established with a 1.22% CV. Due to the rough surface in the layered approximation PMMA insert, the EF is less uniform than that in the smooth 3D CAD insert, which has paraboloid surfaces. However, the layered PMMA insert can still create a uniform and directional EF, with mean EFS of 89.1 mV/mm and a CV of 1.30%.

The double sided tape used in this study is a pressure sensitive adhesive prone to deformation under pressure or stretching. Effect of slight deformation in the chamber thickness to the EF uniformity was examined by numerical simulations. Supplementary Fig. S8 shows the tolerance of mean EFS and CV in chamber thickness ranging from 0.2 mm to 0.3 mm. Although the chamber with a height of 0.26 mm has the smallest CV, all the simulation data of CV are smaller than 3%, demonstrating the versatility of the 3D CAD insert.

The 3D CAD approach can be easily adapted to further scale up the insert for larger petri dishes such as 100 mm TCPS dishes. Uniform EFs are established in both theoretical smooth 3D insert and approximated layered PMMA insert with a height of 10 mm for a 100 mm dish (Table 1 and Supplementary Fig. S9). This demonstrates the flexibility of the 3D CAD principle for creating a uniform EF in a circular shaped device.

### Validation of EF uniformity in the chamber

**Figure 5:**
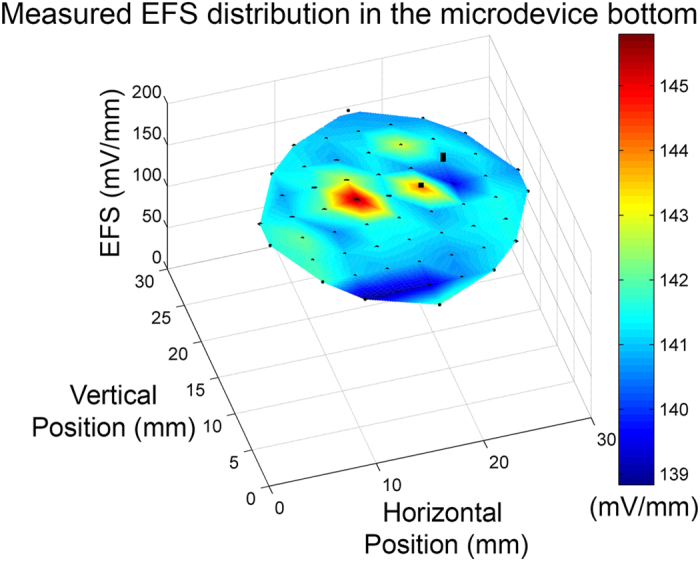
The measured EFS in the insert is shown as the mesh plot and the standard deviations are shown in black error bars. Note that the error in each position is low.

Figure 5 shows the experimentally measured EFS in the bottom chamber by the Ag/AgCl wire electrodes. The EFSs were measured between adjacent holes parallel to the electric current vector. The mean EFS of all measurement is 141.4 ± 1.3 mV/mm with a CV of 0.92%, suggesting that a highly uniform EF is created in the bottom chamber. The 46 V electric potential created an expected 150 mV/mm EFS in the bottom chamber. The measured mean EFS is about 94.3% of the expected value, which coincides with the measurement errors reported in previous studies^29^,^30^. The CV of the measured EFSs is comparable to the 1.30% value expected from numerical simulation (see Table 1). This value is also comparable to the 2.3% CV of measured EFS from the largest rectangular electrical stimulation device reported previously^30^. Finally, the EFSs along the perpendicular direction to the electric current vector is measured to be 4.68 ± 1.90 mV/mm, only 3% of that in the parallel direction.

### High performance cell EF stimulation

While conventional *in vitro* electrical stimulation devices either sacrifice the culture area to stimulate cells uniformly, or stimulate large areas of cells with non-uniform EF, the polymeric circular insert utilised in this study can provide uniform EF stimulation to large area percentage of cells.

The effective stimulation area is defined as the area of the bottom chamber subtracting the area of where the liquid column resides. The effective stimulation area percentage is the ratio of the effective stimulation area over the total surface area of the TCPS dish. The effective stimulation area percentages using the polymeric insert are listed in comparison to those reported in existing literature, see Table 2. Most existing devices cannot achieve uniform EF stimulation in more than 50% of the total cell culture area. Our polymeric inserts can provide uniform EF stimulation in more than 69% of the total area in a 6-well plate (or a 35 mm dish), and up to 90% in a 100 mm petri dish. Thus the cell yields are higher by using polymeric inserts for electrical stimulation. The higher cell yields will greatly benefit subsequent biochemical and molecular biology analysis.

### Cell migration and alignment under uniform EF stimulation

**Figure 6:**
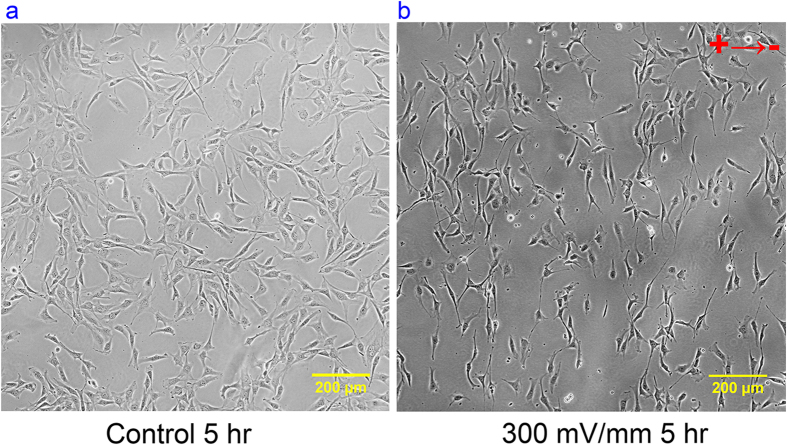
The phase contrast microscopy of NIH/3T3 cells after 5 hours of experiment with the circular polymeric insert: (**a**) without EF stimulation; (**b**) with 300 mV/mm EF stimulation. See more details in SI-Movie-1.

NIH/3T3 fibroblast cells were used to verify the performance of the inserts because they are known to align perpendicular to the EF vector after stimulation and they have shown cathodal electrotaxis^49^,^50^,^51^. The phase contrast microscopy images of the cells under 300 mV/mm EF stimulation over 5 hours were taken and analyzed in Fig. 6 (also see SI-Movie-1).

**Figure 7:**
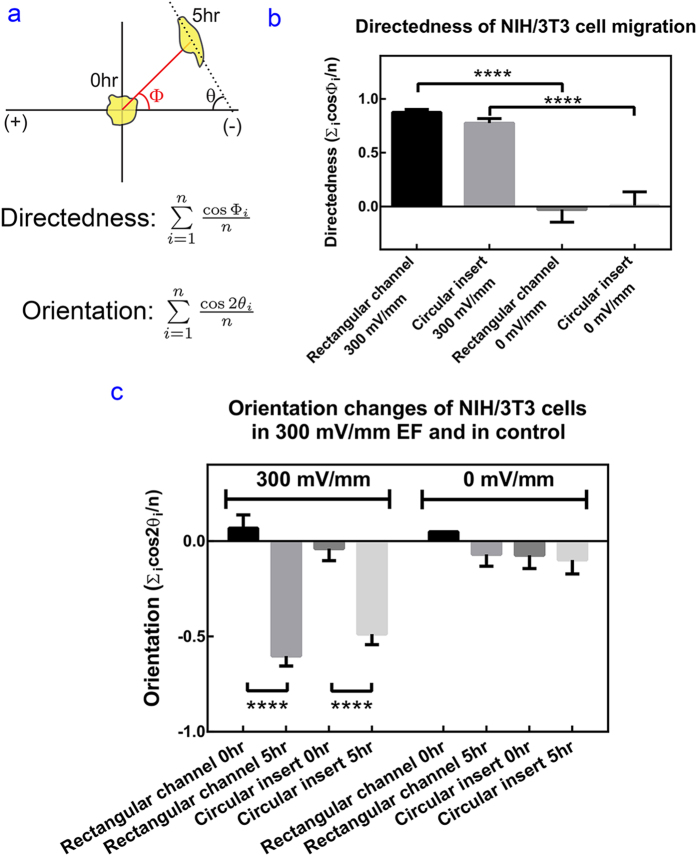
Quantification of the cell migration and alignment measurements. (**a**) Schematic representation of the angle to calculate directedness and orientation. (**b**) Directedness of cell migration in a rectangular channel and circular inserts with and without EF stimulation. (**c**) Cell orientation in a rectangular channel and circular inserts with and without EF stimulation. Four asterisks indicate *p* < 0.0001 from Dunn’s post-hoc test.

To quantify the cell migration and alignment, two parameters (directedness and orientation) are used with the following defintion, see schematics in Fig. 7(a).

- The directedness of cell electrotaxis is defined as the average of 
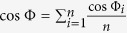
, where Φ_i_ is the angle between the Euclidean vector of each cell migration and the vector of applied EF (from anode to cathode), and *n* is the total number of analyzed cells (see Fig. 7a). A group of anodal moving cells holds a directedness of −1; and a group of cathodal moving cells holds a directedness of +1. For a group of randomly migrating cells, the directedness is zero.
- The orientation is defined as the average of 
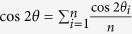
, where *θ*_*i*_ is the angle between the vector of applied EF and the long axis of a given cell; n is the total number of cells analyzed. A group of cells aligned perpendicular to the EF holds an orientation of −1; and a group of cells aligned in parallel to the applied EF holds an orientation of +1. For a group of randomly shaped cells, the average orientation is zero.

The directedness and orientation of the cells with and without EF stimulation are shown in Fig. 7. NIH/3T3 demonstrate strong cathodal electrotaxis under 300 mV/mm EF for 5 hours in both rectangular microfluidic chip and in the circular insert (*p* < 0.0001, in comparison to their respective controls without EF stimulatoin) (Fig. 7b). While the directedness of NIH/3T3 cells in the polymer insert is slightly lower than that in the rectangular channels (0.78 ± 0.02 v.s. 0.87 ± 0.01), there was no statistical significance between the two (*p* > 0.05). This deviation is possibly caused by un-optimised cell culture medium flow rate. While the shear stress in the polymer insert device and the rectangular channel is of the same order, the medium replenishment takes longer for the circular insert due to its bigger cross-sectional area.

Before EF stimulation, cells in both rectangular channels and circular inserts demonstrated random orientation (0.05 to 0.09). After 300 mV/mm EF stimulation, the orientation of cells in rectangular channels and circular insert decreased to −0.60 ± 0.05 and −0.49 ± 0.06, indicating perpendicular alignment (Fig. 7c). The difference of cell alignment in rectangular channels and circular inserts are significant before and after the stimulation (*p* < 0.0001). The control cells in both rectangular channels and the circular inserts do not show any alignment. Detailed cell migration and orientation data is shown in Supplementary Table S1. These results validated the performance of the inserts for electrotaxis experiments comparable to the performance of a rectangular channel. However, the circular inserts have at least two fold higher effective stimulation percentage in comparison to that of rectangular channels, thus more cell yield can be achieved by using our circular inserts.

Moreover, a removable polymeric insert can further aid cell recovery right after the EF stimulation, which can be accomplished by adding a perfluoropolymer-coated layer between the adhesive tape and the insert^30^. Alternatively, the removable insert can be fabricated by using polydimethylsiloxane as the insert material. The silicone rubber can reversibly bond to the TCPS dish with air-tight seal by the clip-on design, similar to those in a transwell insert (Supplementary Fig. S10).

## Conclusion

Establishment of a uniform EF in a circular-shaped microdevice is extremely difficult so the majority of existing EF stimulation devices avoided this issue by using a simple rectangular shaped chamber. The rectangular configuration requires modification to fit with the commercial labware, and only a small portion of the cell culture dish is used for cell culture, thus limiting the cell yield. By adding a 3D CAD based insert in a circular shaped cell culture chamber, we have demonstrated that a uniform EF can be created in a circular-shaped area by modulating the electrical resistance across the device. We highlight our key contributions and outlooks below.

- The effective stimulation area percentage using the insert is at least 2 fold higher than that of existing EF stimulation devices. The yield of cells and its products can be increased for further biochemical analysis.
- The same CAD design principle can be easily scaled up or down to tailor design inserts for different sized TCPS dishes. Mass production of the polymeric insert can be achieved by CNC fabrication, injection molding, or other similar technology. The polymeric insert is useful for adapting electrical stimulation studies in a common laboratory due to the high effective stimulation area percentage and the ease of use.
- The polymeric insert is applicable for various studies. For tissue engineering, EF stimulation has been reported to induce synchronously contracting cardiac tissue^52^,^53^,^54^,^55^,^56^. Osteoblastic differentiation from mesenchymal stem cells can be promoted under EF stimulation^57^. Uniform EF stimulation to circular shaped area could also be useful to stimulate an entire brain slice or tissue slice.

## Additional Information

**How to cite this article**: Tsai, H.-F. *et al*. Uniform electric field generation in circular multi-well culture plates using polymeric inserts. *Sci. Rep*. **6**, 26222; doi: 10.1038/srep26222 (2016).

## Supplementary Material

Supplementary Information

Supplementary Movie S1

## Figures and Tables

**Table 1:** Simulated mean EFS and CV from polymeric inserts with the bottom chamber at the height of 0.26 mm and 0.13 mm.

		0.26 mm thick		0.13 mm thick	
		EFS(mV/mm)	CV	EFS (mV/mm)	CV
6-well	Plain	75.03 ± 5.94	7.91%	74.0 ± 10.06	6.80%
	3D CAD	96.10 ± 1.18	1.22%	96.47 ± 1.38	1.43%
	Layered	89.06 ± 1.63	1.30%	88.92 ± 1.69	1.90%
100 mm	3D CAD	97.44 ± 1.47	1.51%	97.29 ± 2.61	2.68%
	Layered	94.73 ± 1.59	1.67%	94.55 ± 2.72	2.88%

**Table 2:** Stimulation area and effective stimulation area percentage of *in vitro* EF stimulation devices.

Report	Substrate	Thickness (mm)	Stimulation area (cm^2^)	Total area (cm^2^)	Effective stimulation area percentage
Song *et al*.^27^	100 mm TCPS	0.13–0.16	2.2	55	4.0%
Song *et al*.^27^	100 mm TCPS	0.13–0.16	22	55	36.0%
Tandon *et al*.^52^	60 mm glass	0.25	6.5	21	30.9%
Babona-Pilipos *et al*.^58^	60 mm TCPS	0.17	2.2	21	10.5%
Huang *et al*.^29^	150 mm TCPS	0.07	19.2	152	12.6%
Tsai *et al*.^30^	150 mm TCPS	0.6	69	152	45.4%
This study	6-well TCPS;	0.26	6.61	9.5	69.5%
	35 mm TCPS				
	6-well TCPS;	0.13	6.83	9.5	71.9%
	35 mm TCPS				
	100 mm TCPS	0.26	49	55	89.0%
	100 mm TCPS	0.13	49.6	55	90.3%
