# Supplementary material for: Uniform electric field generation in circular multi-well culture plates using polymeric inserts
Source: Sci Rep. 2016 May 19;6:26222. doi: 10.1038/srep26222 (PMC4872143; doi:10.1038/srep26222)
Supplement: Supplementary Information [file srep26222-s1.pdf]

Uniform electric field generation in circular multi-well culture plates using polymeric inserts

Hsieh-Fu Tsai[1], Ji-Yen Cheng[2], Hui-Fang Chang[2], Tadashi Yamamoto[3], Amy Q. Shen[1]

[1] Micro/Bio/Nanofluidics Unit, Okinawa Institute of Science and Technology Graduate University, Okinawa, Japan
[2] Research Center for Applied Sciences, Academia Sinica, Taipei, Taiwan
[3] Cell Signal Unit, Okinawa Institute of Science and Technology Graduate University, Okinawa, Japan

May 5, 2016

1 Supplementary Figures and Tables

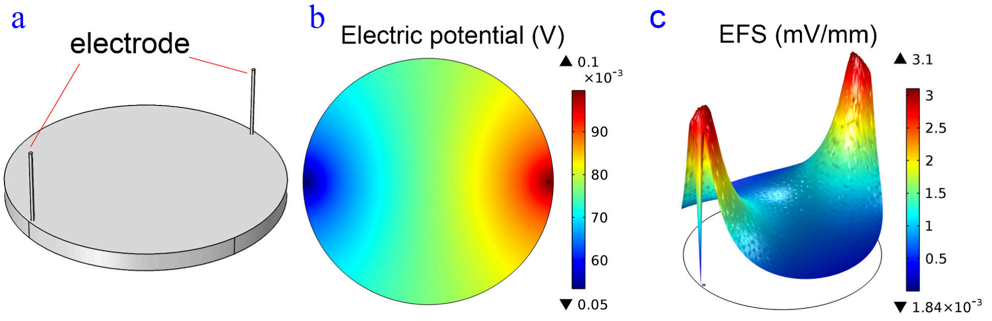

Fig. S1: Numerical simulation of an electric field (EF) stimulation device designed by Marotta et al. [1]. (a) The 3D model consists of a 2 mm-deep bottom chamber in a 6-well plate and two thin platinum electrodes inserted on the opposite sides of the chamber. (b) The electric potential distribution of the EF. The non-uniform decrease of the electric potential between the electrodes creates a non-uniform EF. (c) With an electrical current at 500 A/m^2 introduced from one electrode and flows to the other at ground potential, a wide distribution of the electric field strength (EFS) in the bottom chamber renders non-uniform EF.

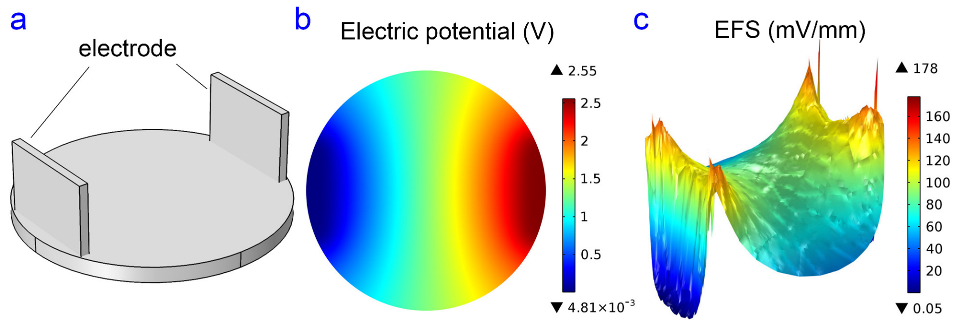

Fig. S2: Numerical simulation of a commercial C-dish EF stimulation device. (a) The 3D model consists of a 2 mm-deep bottom chamber in a 6-well plate and two carbon plate electrodes inserted on the opposite sides of the chamber. (b) The electric potential distribution of the EF. The non-uniform decrease of the electric potential between the electrodes creates a non-uniform EF. (c) The EF distribution at the bottom of the chamber is generated by 500 A/m^2 electrical current flowing from one electrode to the other at ground potential. The non-uniformity of the EF is evident by the rough surface of the EFS distribution in the bottom chamber.

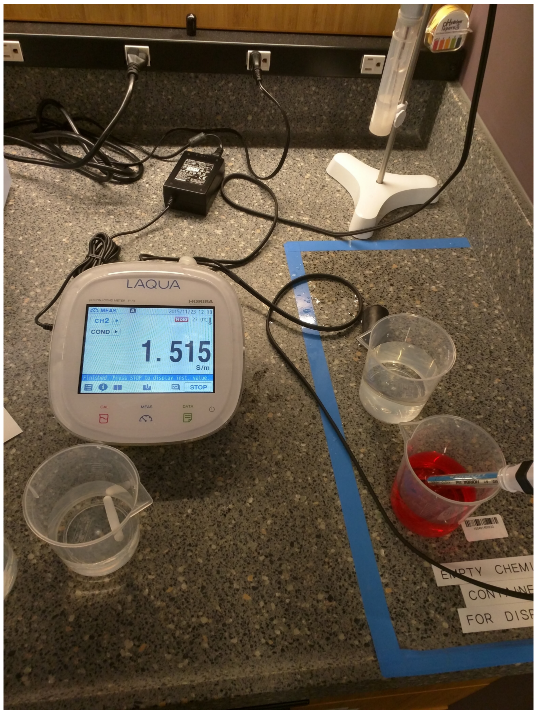

Fig. S3: A photo snapshot of the conductivity measurement setup. The conductivity of Dulbecco's minimum essential medium was measured by using a calibrated conductivity probe.

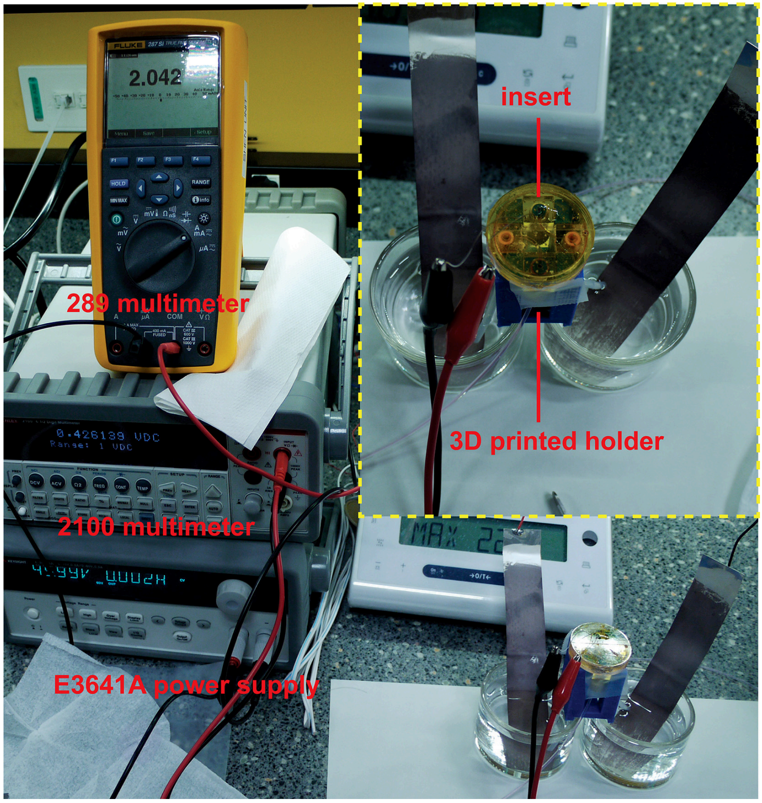

Fig. S4: A photo snapshot of the measurement setup. The inset shows a magnified image of a PMMA insert on a custom 3D printed holder for EF measurement.

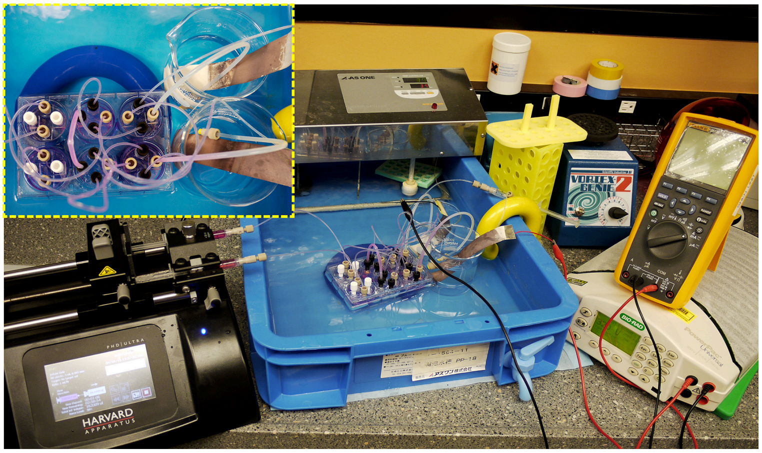

Fig. S5: A photo snapshot showing the setup of EF stimulation of cells on a 6-well plate in a temperature controlled water bath. Fresh cell culture medium was infused by a syringe pump (Harvard Apparatus, USA). The DC electric potential bias to create an EF was applied by a BioRad DC power supply.

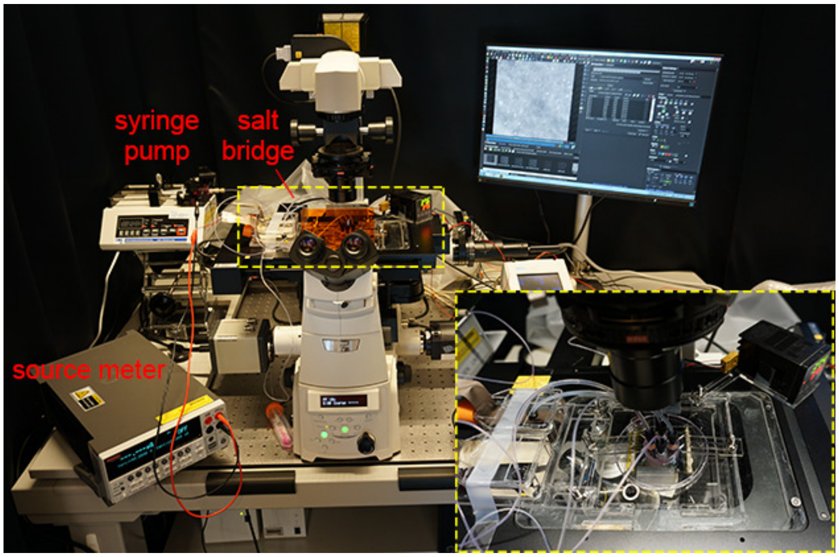

Fig. S6: A photo snapshot showing the microscopy setup for time lapse cell electrotaxis experiment. The device was placed on top of a transparent heater on the microscope stage to provide temperature control for cell survival. Continuous culture medium was pumped through a syringe pump. The direct current electrical stimulation and current monitoring was applied through a source meter. The inset shows how the devices were fixed on the microscope stage.

Table. S1: Quantitative analysis of cells tracked in time-lapse electrotaxis experiments

	N	directedness	SEM	orientation 0hr	SEM	orientation 5hr	SEM
300mV/mm rectangular channel	106	0.8753	0.014	-0.07	0.069	-0.60	0.053
300mV/mm circular insert	110	0.7758	0.021	-0.04	0.062	-0.49	0.056
Control rectangular channel	118	-0.03	0.059	-0.05	0.067	-0.07	0.062
Control circular insert	103	0.01	0.064	-0.07	0.069	-0.09	0.07

N: number of cells analyzed; SEM: standard error of mean.

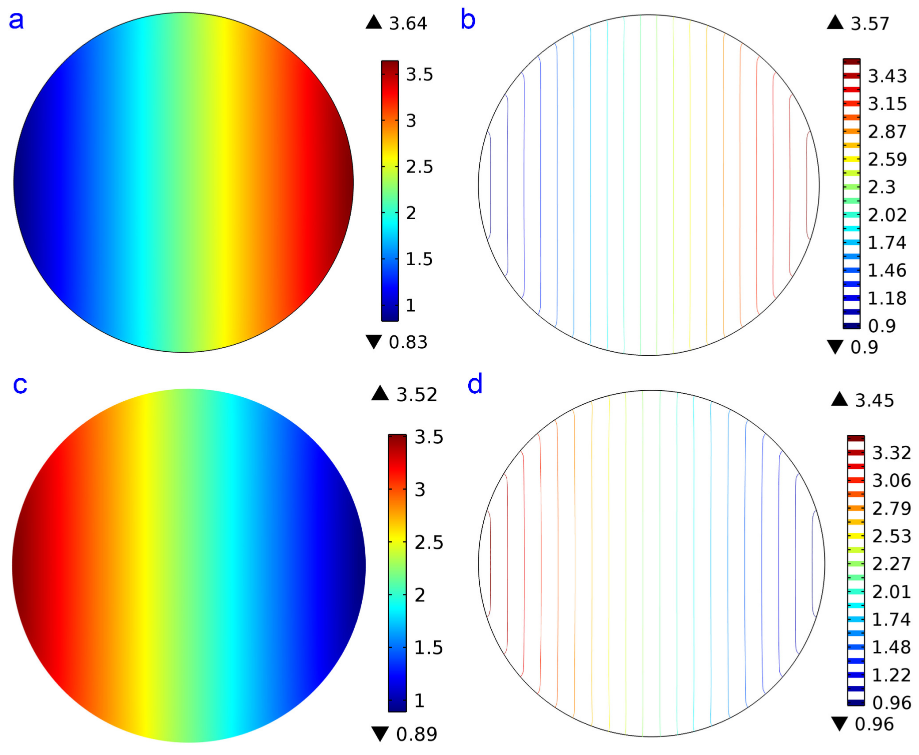

Fig. S7: The electric potential distribution in the 3D CAD polymeric insert designed in this study. The uniform distribution of electric potential yields the uniform EF in the bottom chamber. Electric potential distribution shown as (a) surface plot and (b) contour plot at the bottom of the insert with smooth 3D CAD structures. Electric potential distribution shown as (c) surface plot and (d) contour plot at the bottom of a 6-layered PMMA insert. Both inserts with smooth 3D CAD structures and the layered PMMA insert can create a uniform and directional EF. The directionality of the EF can be reversed by placing the high voltage to the opposite salt bridge as shown in (a) and (c).

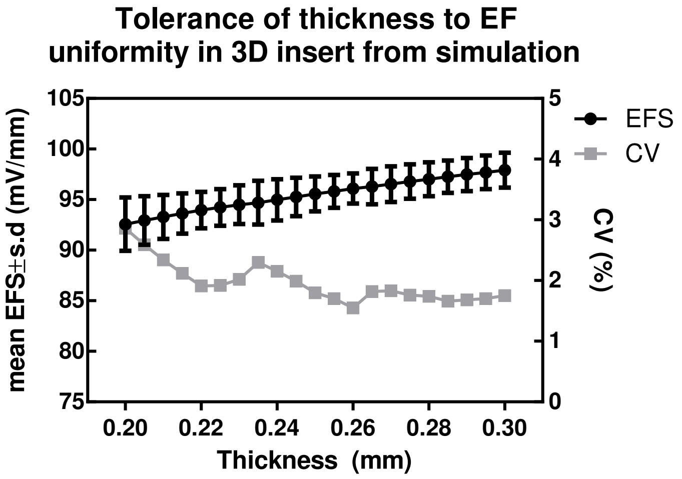

Fig. S8: Tolerance of EF uniformity to the thickness of the chamber in a CAD insert, examined by numerical simulations. By increasing the thickness of the bottom chamber thickness from 0.2 mm to 0.3 mm, the resistance decreases and the mean EFS slightly increases (see dark circles). But the simulated mean EFSs in the chamber with this thickness range are very close to the intended value of 100 mV/mm. The coefficient of variation (CV) describing the non-uniformity in the EFS is the lowest in the intended 0.26 mm-thick chamber. Nevertheless the CVs in all thickness examined are below 3 %, verifying the robustness of the CAD insert for creating a uniform EF (see light squares).

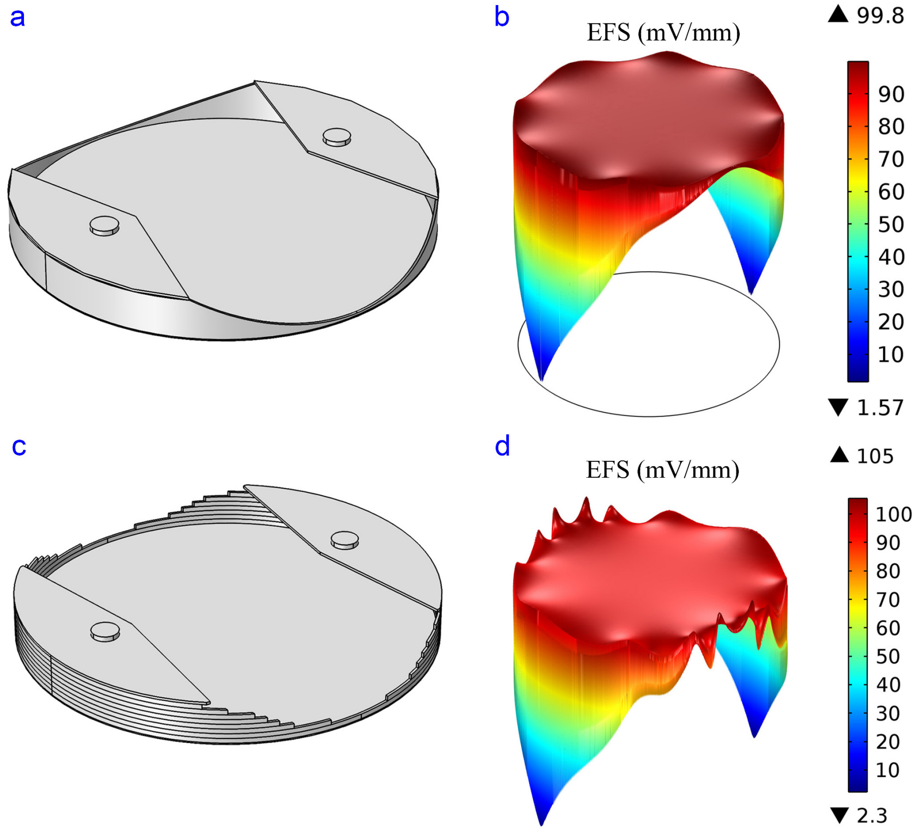

Fig. S9: Numerical simulations of a scaled-up insert for a 100 mm tissue culture polystyrene dish. (a) CAD model for the insert with paraboloid surfaces; (b) The uniform EF created in (a); (c) The 6-layered PMMA insert; (d) The uniform EF created in (c). In the smooth 3D CAD model, the mean EFS and the CV of the EFS are 97.44±1.47 mV/mm and 1.51 % in (b). The mean EFS and the CV of the EFS in the layered insert (d) are 94.73±1.59 mV/mm and 1.67 %. The layered inset is less ideal than the smooth 3D CAD model due to its rough surfaces but it does not effect the uniformity of the EF stimulation.

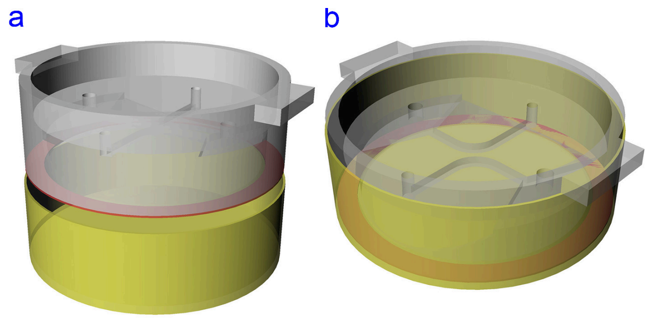

Fig. S10: An easy to use insert based on reversible bonding silicone pad (pink) that is compatible with mass production process (a) before insertion; (b) after insertion. Pressure can be applied on the insert to seal the chamber by a 0.26 mm thick elastomeric silicone O-ring.

2 Design principle of a 3D CAD insert to create a uniform electric field (EF)

In contrast to conventional *in vitro* EF stimulation systems using a rectangular chamber, to apply a uniform EF in a circular shaped chamber (i.e., tissue culture polystyrene petri dish) is extremely difficult. Here we show the design principle of a 3D CAD insert in a microfluidic system, with the aim to create a uniform EF in the bottom chamber where cells are cultured and stimulated (Fig. S11).

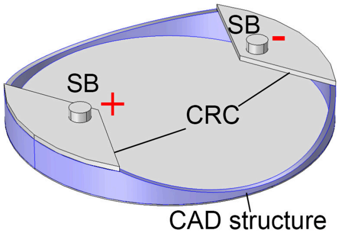

Fig. S11: Schematic representation of a 3D model for creating the uniform EF in an assembled microfluidic chip. CRC stands for current rectifying chambers. SB stands for salt bridge. CAD structures are colored in blue.

To maximize the cellular products for biochemical analysis, we aim to use as much of culture area as possible to increase the amount of cells to be stimulated in the circular chamber with a given height (red, Fig. S12). The electric field will be applied through a thin layer of fluid volume surrounding the perimeter of the bottom chamber through an insert, and the fluid volume can be treated as a thin liquid column (LC, purple block in Fig. S12). However, a uniform EF cannot be created by applying two electric potentials on top of this liquid column.

To create a uniform EF, the electrical resistance from one electrode to the other through any cross-section in the system must be the same. To address this challenge, we propose a three dimensionally (3D) designed structure to equalize the electrical resistances through any arbitrary current line that passes through the bottom circular chamber. Such structure can be approximated as the liquid column (LC) intersected by two identical circular paraboloids (P_1&P_2) at the intersection of midpoint between the two potentials and the diameter of the liquid column (Fig. S12). The 3D CAD structure (green block, Fig. S12) to equalize the electrical resistance can be created by trimming the liquid column using the circular paraboloids. Boolean difference tool was used in the CAD software to carry out this step. The 3D CAD model can then be used to create a uniform EF in the assembled microfluidic chip.

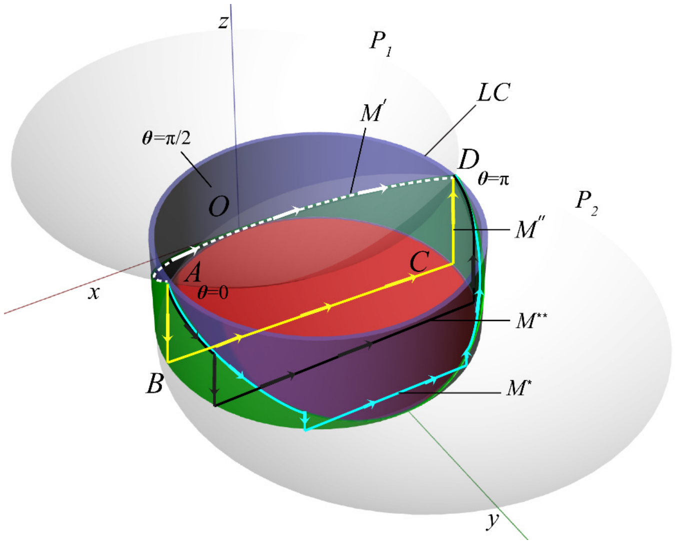

Fig. S12: Geometrical presentation of the design principle. Two identical axial symmetric paraboloids (P_1&P_2) along $\overline{BC}$ are used to equalize the electrical resistance in the bottom chamber (red region) by trimming the liquid column LC (purple region).

According to Ohm's law, electrical resistance R can be described as [2]

$$R = \rho \frac{l}{A_{\text{cross-section}}}, \tag{1}$$

where ρ is the resistivity, l is the length, and $A_{\text{cross-section}}$ is the cross-sectional area of the conductor. We need to identify the arc length of the curves in our system to calculate the electrical resistance. Using a polar coordinate system, the bottom chamber (Fig. S12, red circle) resides on the xy plane and the liquid column on top possesses a diameter of $\overline{BC}$. For paraboloid P_1, its apex resides with the origin O in the liquid column and the yz plane. The paraboloid intersects with the liquid column at the curve $M' = \widehat{AOD}$ (white dashed line, Fig. S12). The projections of points A and D on xy plane are B and C. Consider an electrical current flowing from point A to point D. By Ohm's law, in order to have a uniform EF in the chamber bottom, the resistance of $\widehat{AOD}$ (Fig. S12, white dashed path) must be equal to that of $\overline{AB} + \overline{BC} + \overline{CD}$ (Fig. S12, yellow path), equivalently, $R_{M'} = R_{\overline{AB}} + R_{\overline{BC}} + R_{\overline{CD}}$. If we assume the resistivity is the same across the sample,

$$\frac{\widehat{AOD}}{A_{\text{M'}}} = \frac{M'}{A_{\text{M'}}} = \frac{\overline{AB}}{A_{\text{AB}}} + \frac{\overline{BC}}{A_{\text{BC}}} + \frac{\overline{CD}}{A_{\text{CD}}}. \tag{2}$$

Equation 2 shows that a uniform EF distribution can be enabled when the ratio of the rectification of the curve M' over the cross-sectional area of the liquid column equals to the ratio of $\overline{AB} + \overline{CD}$ over the cross-sectional area of the liquid column, in addition to $\overline{BC}$ over the cross-sectional area of the bottom chamber. Assuming all electrical currents pass through sufficiently thin paths,

$$\frac{M'}{\text{column thickness}} = \frac{2 \times \overline{AB}}{\text{column thickness}} + \frac{\overline{BC}}{\text{chamber depth}}. \tag{3}$$

Since $\overline{AB}$ and $\overline{BC}$ can be easily measured, the value of M' can be subsequently extracted by using Equation 3. With the information of M', we can determine the shape of the circular paraboloid P_1 and P_2 as follows.

The shape for the circular paraboloid P_1 and P_2 can be described as

$$\frac{x^2 + y^2}{a^2} = \frac{z}{b}, \tag{4}$$

where a^2/b is the constant describing the level of curvature in xz and yz planes of the paraboloid, which defines the shape of the paraboloid.

The equation for the liquid column can be considered as

$$x^2 + (y - r)^2 = r^2, \tag{5}$$

where r is the radius of the column (also the radius of the circular bottom chamber).

The coordinates of points O, A, and D can be presented in parametric form $\left(r\cos\theta, r(1-\sin\theta), \frac{2br^2}{a^2}(1-\sin\theta)\right)$, with

$$O : \left(r\cos\frac{\pi}{2}, r(1-\sin\frac{\pi}{2}), \frac{2br^2}{a^2}\left(1-\sin\frac{\pi}{2}\right)\right) = (0, 0, 0), \tag{6a}$$

$$A : \left(r\cos 0, r(1-\sin 0), \frac{2br^2}{a^2}(1-\sin 0)\right) = \left(r, r, \frac{2br^2}{a^2}\right), \tag{6b}$$

$$D : \left(r\cos\pi, r(1-\sin\pi), \frac{2br^2}{a^2}(1-\sin\pi)\right) = \left(-r, r, \frac{2br^2}{a^2}\right). \tag{6c}$$

The rectification of curve M' along the paraboloid P_1 can be calculated by integrating parametric equations [3],

$$M' = \int_0^\pi \sqrt{\left(\frac{dx}{d\theta}\right)^2 + \left(\frac{dy}{d\theta}\right)^2 + \left(\frac{dz}{d\theta}\right)^2} d\theta \tag{7a}$$

$$= \int_0^\pi \sqrt{(-r\sin\theta)^2 + (-r\cos\theta)^2 + \left(-\frac{2br^2}{a^2} \times \cos\theta\right)^2} d\theta \tag{7b}$$

$$= \int_0^\pi \sqrt{r^2 + \left(\frac{2br^2}{a^2}\right)^2 - \left(\frac{2br^2}{a^2}\right)^2 \sin^2\theta d\theta}. \tag{7c}$$

The constant of the paraboloid $\frac{a^2}{b}$ can be solved by solving Equation 3. Equation 3 is difficult to solve explicitly due to the elliptic integral of the second kind related to M' along the paraboloid P_1 (Equation 7). But Equation 3 can be evaluated using Mathematica with the following code (Listing S1) by denoting a constant $c = \frac{2br^2}{a^2}$ (r is the radius of the circular bottom chamber) to simplify the calculation.

Listing S1: Example code to find the descriptor for the paraboloid P_1

```
Plot[Evaluate[Integrate[Sqrt[225+c^2-c^2*Sin[x]^2,{x,0,Pi}]/0.5-2*c
/0.5-30/0.26],{c,-4.715,-4.71}]
```

To design an insert for 6-well plates, denote the thickness of the liquid column to be 0.5 mm, the diameter of the bottom chamber to be 30 mm, and the thickness of the bottom chamber to be 0.26 mm, the parameter $c = \frac{2br^2}{a^2}$ (containing the constant for the paraboloid P_1) can be evaluated and used to create the 3D model (described below) in a commercial CAD software for further numerical simulation and device fabrications.

$$\frac{b}{a^2} = -0.01047. \tag{8}$$

$$P_1 : x^2 + y^2 = -\frac{z}{0.01047}. \tag{9}$$

With this design principle we can consider two extreme cases: electrical current passing through the shortest chord and the longest chord (i.e., diameter), which will yield the constant of the paraboloid and the height of the liquid column required to achieve uniform EF. To find the electrical resistance for all arbitrary current lines passing through each different paths (for example, pathline curves M'', M^{**}, M^{*}, and M', see Fig. S12) between the shortest chord ($\theta = 90°$) and the diameter ($\theta = 0°$), where θ is the polar coordinate azimuth, the following equation in listing S2 can be used.

Listing S2: examples code for all M paths

```
Table[Evaluate[2*Integrate[Sqrt[225+4.713^2-4.713^2*Sin[x]^2],
{x,0,y}]/0.5+2*(-4.713)*(1-Sin[y])/0.5+2*15*Sin[Pi/2-y]/0.26], {y,0,Pi/2, Pi/180}]
```

The calculated resistance range with $\theta \in (0°, 90°)$ is plotted in Fig. S13. The relative resistance of $\overline{AB}+\overline{BC}+\overline{CD}$ corresponds to $\theta = 0°$ and that of $\widehat{AOD}$ corresponding to $\theta = 90°$. The results show that resistances of all path lines converge toward both extreme cases and our current model provide a good approximation to achieve uniform EF in a circular chamber. For future work, we plan to use quadratic surface integral to further improve our design.

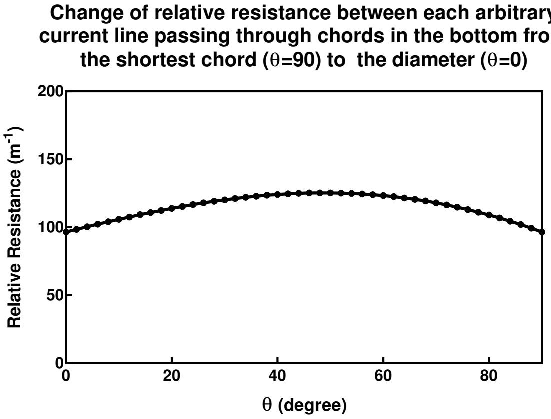

Fig. S13: The change of relative resistance between each arbitrary current line passing through each chord in the bottom chamber from the shortest chord to the diameter, by varying θ from 0° to 90°.

3 Supplementary Movie

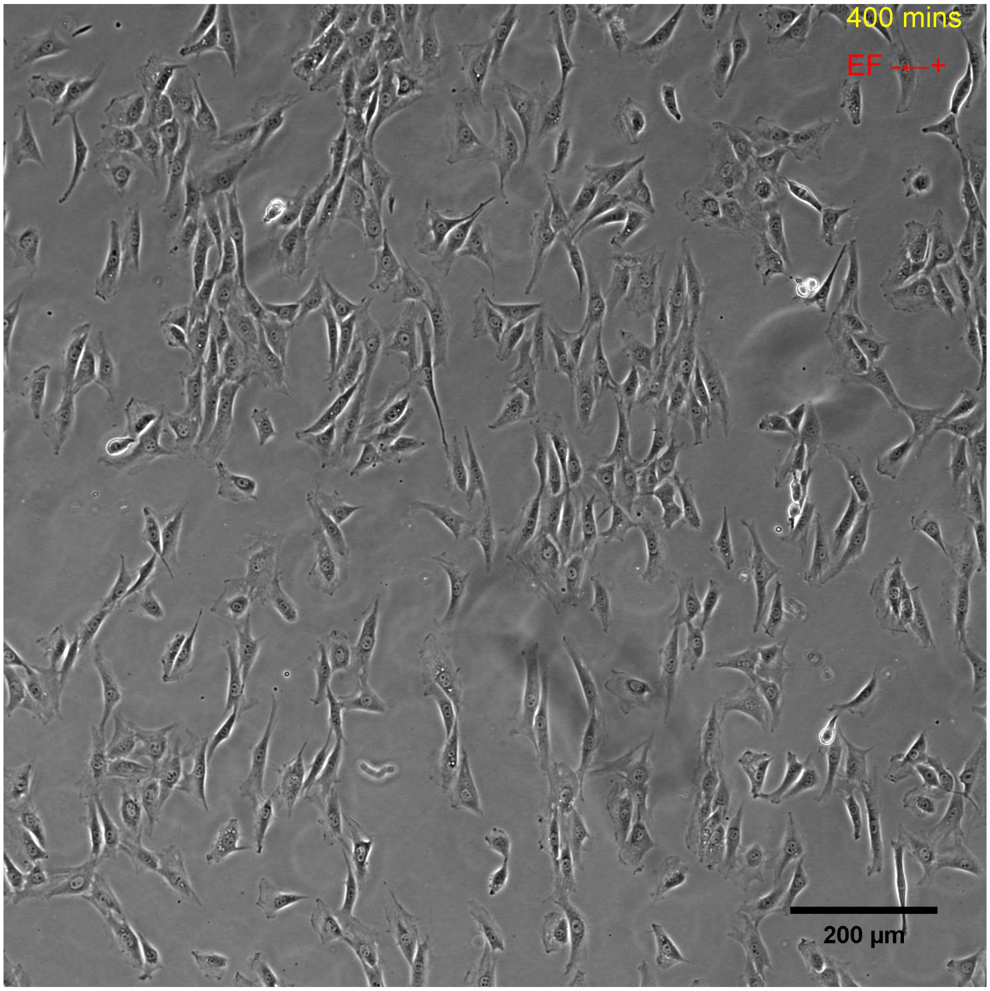

Movie. S1: A supplementary movie showing the the electrotaxis and electroalignment of NIH/3T3 cells under 300 mV/mm EF under time lapse phase contrast microscopy.

References

[1] Marotta, M., Bragós, R. & Gómez-Foix, A. M. Design and performance of an electrical stimulator for long-term contraction of cultured muscle cells. *BioTechniques* **36**, 68–73 (2004).

[2] Hobbie, R. K. & Roth, B. J. *Intermediate Physics for Medicine and Biology* (Springer, 2015).

[3] Larson, R. & Edwards, B. H. *Calculus Multivariable* (Cengage Learning, 2009).
